# Optimization of Biologically Inspired Electrospun Scaffold for Effective Use in Bone Regenerative Applications

**DOI:** 10.3390/polym16142023

**Published:** 2024-07-15

**Authors:** Susai Mani Mary Stella, Murugapandian Rama, T. M. Sridhar, Uthirapathy Vijayalakshmi

**Affiliations:** 1 Department of Chemistry, School of Advanced Sciences, Vellore Institute of Technology, Vellore 632014, India; stellasaren23@gmail.com; 2 Centre for Nonlinear Systems, Chennai Institute of Technology, Chennai 600069, India; ramapandianchemist@gmail.com; 3 Department of Analytical Chemistry, Guindy Campus, University of Madras, Chennai 600025, India; tmsridhar23@gmail.com

**Keywords:** hydroxyapatite, electrospinning, biomineralization, cell viability, bone tissue regeneration

## Abstract

Human bone is composed of organic and inorganic composite materials, contributing to its unique strength and flexibility. Hydroxyapatite (HAP) has been extensively studied for bone regeneration, due to its excellent bioactivity and osteoconductivity, which makes it a highly valuable biomaterial for tissue engineering applications. For better therapeutic effects, composite nanofibers containing polyvinyl alcohol (PVA) and polyvinyl Pyrrolidone (PVP) were developed using an electrospinning technique in this study. Herein, hydroxyapatite (a major inorganic constituent of native bone) concentrations varying from 5 to 25% were reinforced in the composite, which could alter the properties of nanofibers. The as-prepared composite nanofibers were characterized by SEM, TEM, XRD, and FT-IR spectroscopy, and a bioactivity assessment was performed in simulated body fluid (SBF). The ICP-OES analysis was used to determine the concentration of Ca^2+^ and PO_4_^2–^ ions before and after SBF immersion. To optimize the material selection, the nanofibrous scaffolds were subjected to cell proliferation and differentiation in MG-63 osteoblast cell lines, but no significant toxicity was observed. In conclusion, HAP-PVA-PVP scaffolds exhibit unique physical and chemical properties and ideal biocompatibility, with great promise to serve as effective candidates for bone tissue applications.

## 1. Introduction

Tissue engineering is a crucial resource for addressing diagnostic and therapeutic concerns associated with clinical manifestations, such as bone replacement, wound dressing, drug delivery, bone defects, cartilage implantation, and other biomedical applications [1,2]. Bone tissue engineering is an emerging arena to inculcate regenerative therapies for bone damage and disease. Human bone is composed of both organic and inorganic material, which supports the structural integrity and functionality of the internal system. The bone’s organic components are polysaccharides and type 1 collagen, which provide flexibility/tensile strength to the bone [3]. The inorganic bone mineral, in particular, the carbonated hydroxyapatite (HAP) (Ca_10_ (PO_4_)_6_(OH)_2_), promotes stiffness/compressive strength for bones [4,5]. Common synthetic methods used to prepare HAP include the wet chemical precipitation method, sol–gel method, mechanochemical synthesis, microwave-assisted synthesis, solid-state reaction, and hydrothermal. HAP is also recognized as an excellent material choice for dental applications due to the fact that it is applied directly to bone tissue and promotes bone tissue growth [6,7,8,9,10]. However, synthetic HAP has a few disadvantages, such as its hard brittle nature, relatively low tensile strength, limited load-bearing properties, and slow degradation, unless combined with other materials. To overcome this problem, polymers are often used as a supporting material to expand its mechanical strength and degradation properties [11,12]. A variety of polymers, both natural and synthetic, have been used in the fabrication of scaffolds, considering the various properties that can aid in better bone regeneration [13,14,15,16]. Amongst these, PVA and PVP have excellent material properties in biomedical applications. PVA has exceptional features, such as good biocompatibility, suitable degradability, good chemical resistance, and hydrophilicity [17]. In addition, PVP is hemocompatible, nontoxic, and has better blending abilities with other polymers as well as inorganic moieties, to form a composite material. The hydroxyl group of PVA and proton-accepting carboxyl groups of PVP interact with each other to form a polymeric scaffold with good mechanical properties and controlled solubility [18,19].

The bone regeneration ability of the fabricated composite scaffold consisting of bioactive polymers and ceramics is studied in simulation body fluid to assess the differentiation and proliferation of osteoblasts for artificial bone formation, as it resembles human bone, which is made of a combination of an apatite layer and natural polymer [20]. It has been proved that a scaffold with an appropriate pore volume and size can act as a basal plate for cell interaction, and it can provide structural support for cell adhesion and migration, including mechanical properties where bone repair sites are ready for new tissue formation [21]. However, if the pore size is too large, the cell adhesion capacity would be limited due to the insufficient surface area. In contrast, a less-than-sufficient pore size can result in limited migration and proliferation, which can cause a negative impact on tissue formation/functions [22]. To overcome this problem, the optimization of appropriate scaffold fabrication is essential for successful tissue formation. Various methods have been used for the fabrication of scaffolds that can enhance cell growth. These discrete methods include freeze drying, electrospinning, solvent casting/particulate leaching, fiber bonding, gas foaming, melt molding, porogen leaching, space holder, self-assembly methods, and 3D-printing techniques, which are suitable for different biomedical applications [23,24,25,26].

The electrospinning technique stands high among other techniques due to its dexterity in fabricating fibers at the nanoscale level, with an easy functionalizing potential, making it ideal for biomedical applications [27]. Desired fiber diameters can be achieved by controlling parameters, such as viscosity, conductivity, surface tension, tip-to-collector distance, and electric field strength. In electrospinning, a high electric field is applied to melt the solution (a droplet of the fluid obtained at the edge of the needle), which acts as a positive electrode. Once the solution is melted, it leads to the deformation of droplets and the ejection of a charged jet from the tip of the needle, accelerating toward the negative electrode connected to the collector [28]. In this technique, there is still a big challenge to develop an electrospun sheet of sufficient thickness by ensuring the physical, chemical, and structural blending of the ceramics and polymer composite [29].

In the present investigation, nanofibers containing PVA and PVP were fabricated using the electrospinning technique. Hydroxyapatite (HAP) concentrations of 5%, 10%, 15%, 20%, and 25% were included to vary the mechanical properties, biodegradability, and bioactivity of the fabricated nanofibers. The chemical composition of the nanofibers was characterized by means of FT-IR and XRD to recognize the functional groups and phase purity. The thermal stability of the nanofibers in the presence as well as absence of HAP was examined by means of TGA and DSC. The surface morphology and fiber diameter were analyzed by SEM and TEM, and the influence of the nanofibrous compositions in terms of bioactivity was analyzed by means of SBF immersion studies. The ICP-OES analysis was expended to establish the concentration of Ca^2+^ and PO_4_^3–^ ions present in the SBF solution. The surface area, pore size, pore volume, and elemental ratio were analyzed by means of BET and XPS studies. Finally, the nanofibrous compositions were subjected to cell proliferation and cell growth in MG63 osteoblast cells.

## 2. Materials and Methods

Commercialized polymers such as PVA with a molecular weight of approximately 85,000–124,000 g/mL (Sigma-Aldrich, Bangalore, India) and PVP (K-90) were selected for the fabrication of nanofibers. Ammonium dihydrogen orthophosphate (NH_4_H_2_PO_4_), calcium nitrate tetrahydrate (Ca(NO_3_)_2_.4H_2_O), and aqueous ammonia were used for the preparation of HAP.

### 2.1. Preparation of Hydroxyapatite

Thus, 1 M of calcium nitrate tetrahydrate and 0.6 M of ammonium dihydrogen orthophosphate were prepared using double-distilled water. Phosphate solution was added dropwise into the calcium solution under stirring conditions. Finally, the solution pH was adjusted to 11 using aqueous NH_3_. The solution was subjected to aging for 16 h. The raw HAP was sintered at 900 °C for 2 h to attain phase purity. The sintered composite was finally powdered using mortar and pestle; further, the powder was sieved using a 25 µm test sieve for the development of nanofiber (MESH.NO. 500).

### 2.2. Electrospinning of Polymer Scaffolds

The fabrication of nanofibers using solution containing 5 wt% of PVA and 5 wt% of PVP with various concentrations of HAP (5, 10, 15, 20, and 25 wt%) is shown in Table 1. Initially, PVA and PVP solution was made using 0.5 g of PVA and 0.5 g of PVP dissolved in 10 mL of double DW (distilled water) under stirring conditions at 80 °C for 2 h. HAP synthesized by co-precipitation method was added in different weight ratios such as 5, 10, 15, 20, and 25 wt% in polymer compositions of PVA/PVP under stirring conditions and aged for 12 h. The developed solution was loaded onto a syringe needle (5 mL) and was spun on the collector (grounded aluminum foil; speed 700 rpm; voltage 25 kV; flow 0.5 mL; distance 15 cm). Finally, the mats were dried in a hot air oven at 70 °C for 24 h.

### 2.3. Characterization Techniques

Prepared electrospun nanofibers were examined using a Fourier-Transform Infrared spectrometer (IR Affinity-1, SHIMADZU Corporation, Kyoto, Japan in a wavenumber range from 400 to 4000 cm^−1^). The SHIMADZU X-ray diffractometer (model Lab XRD- 600, Kyoto, Japan) with Cu Kβ radiation (λ) = 1.5418 Å at the 2θ scan range of 0° to 90°), scanning electron microscopy (FE-SEM: JEOL instrument, Quanta, FEG 250, Akishima-shi, Japan, at a test voltage 10–20 kV), transmission electron microscopy (TEM, JEOL, Welwyn Garden City, UK, at an accelerating voltage of 200 kV), X-ray photoelectron spectroscopy (XPS, ESCALAB 250Xi, Richardson, TX, USA) were used to analyze material surface chemistry. Thermogravimetric analysis (TGA) and differential scanning calorimetry (DSC equipment model SDT Q600 V20.9 Build 20, New Castle, RT > 1200 °C @ 20 °C/min N2 Purge = 100 mL) were also used.

### 2.4. Bioactivity Study Using SBF Immersion

Using SBF solution, the bioactivity was examined for prepared samples, and the solution was prepared according to Kokubo et al., 1990 [30]. After the addition of all the chemicals, the solution pH was maintained around 7.3 using tris buffer. The prepared nanofibers were immersed in the SBF solution with different periods, and every 24 h, the solution was refreshed. Finally, the nanofibers were analyzed by SEM-EDAX, JSM-5610LV, JEOL, Japan) to confirm the formation of apatite on the surface of the scaffold. Each sample was mounted onto an electronically conducting carbon tape on aluminium stubs before being gold-coated at an accelerating voltage of 10 kV and 5 kV.

### 2.5. Hemocompatibility Analysis

The blood compatibility of the prepared electrospun nanofibers was evaluated by hemolytic assay (ASTM F 756-00 guidelines). Human red blood cells were separated from plasma, the scaffolds were sterilized, and about 20 µL of diluted blood (20 µL of fresh anticoagulant blood in 10 mL 0.9% saline) was dropped into 5 mL sample vials containing nanofibers. Samples were incubated at 37 °C for 60 min and centrifuged at 2500× *g* rpm. The hemolytic percentage was determined using UV-visible spectrophotometry (Shimadu UV-1800, Kyoto, Japan), and the values were recorded at a wavelength of 545 nm. The hemolysis ratio (HR) was calculated as follows [31],

$$HR=\frac{Dt-Dnc}{Dpc-Dnc}\times 100\%$$

where HR is the hemolysis ratio, and Dt, Dnc, and Dpc are average absorbance values of the respective sample, negative control (saline), and positive control (distilled water); the HR values between 0 and 2 are considered as non-hemolytic, whereas values between 2 and 5 are considered as slightly hemolytic.

### 2.6. Alkaline Phosphatase Assay

Alkaline phosphatase activity (ALP) activity was investigated for 24 h and 48 h, and 100 μL of RIPA (radioimmunoprecipitation assay) buffer solution was added to the cell culture medium containing the scaffold and control samples to remove the proteins from the cell. The contents of the wells were centrifuged (12,000× *g* rpm) at 4 °C and, and 1 μL of (p)-nitrophenol phosphate solution was added to each sample and measured by an ELISA reader at a wavelength of 450 nm.

### 2.7. Biocompatibility Assessment

In vitro cell viability of the prepared nanofibers was investigated using MG63 human osteoblast cell line using 96-well plates. The solution and 10% fetal bovine serum (FBS, Himedia, Mumbai, India) were used in a controlled atmosphere (5% CO_2_; T = 37 °C), and the cells were washed with 200 μL of 1X PBS, treated with various concentrations of the synthesized scaffolds in serum-free media and kept for incubation overnight. MTT solution at a concentration of 0.5 mg/mL was then added and incubated, and the medium was discarded from the cells and washed using PBS. Color intensity was monitored using formazan dye’s for each well plate and was measured at 570 nm using a microplate spectrophotometer. The percentage of cell viability was produced with respect to OD values of control using the following calculation [32]:

Cell viability%=Intensity of sampleIntensity of control×100

## 3. Results

### 3.1. FT-IR Analysis of HAP-PVA-PVP Nanofibers

**Figure 1:**
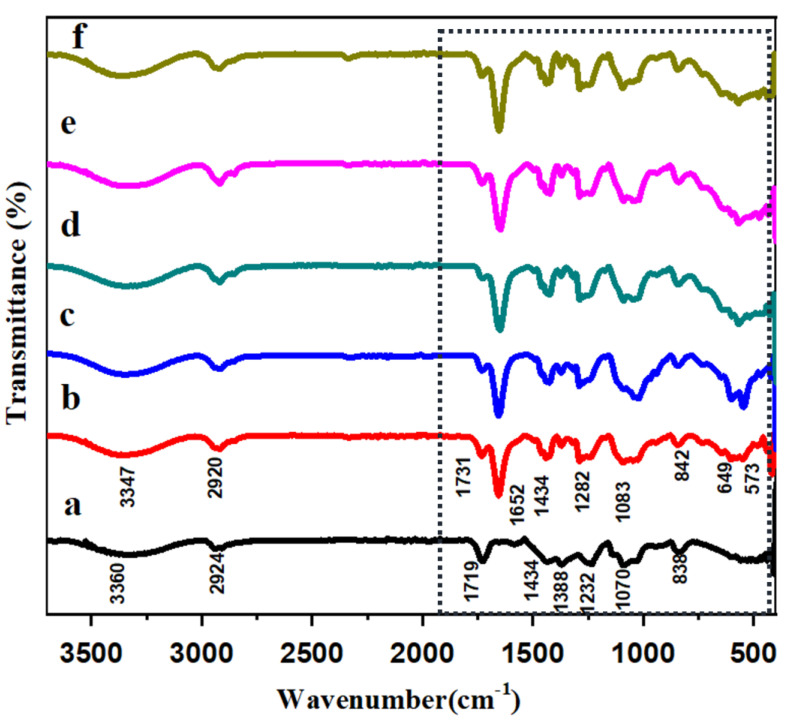
FTIR analysis of (**a**) PVA-PVP; (**b**) 5%; (**c**) 10%; (**d**) 15%; (**e**) 20%; and (**f**) 25% of HAP-PVA-PVP.

The FT-IR spectrum (Figure 1) shows prominent bands of HAP along with the bands of PVA and PVP in the fabricated nanofibers. In Figure 1a, (PVA-PVP only) represents the C-H bending at 838 cm^−1^ in PVA polymer; the band at 1070 cm^−1^ confirms the presence of C-O vibration of polymer [33]. A band at 1232 cm^−1^ is attributed to the C-N bond, mainly from the functional group of PVP. The characteristic band at 1383 cm^−1^ confirms the C-OH band in the polymeric network. The characteristic small bands at 1434 and 1719 cm^−1^ were ascribed to C=O stretching of PVP. The band at 2924 cm^−1^ was assigned as the asymmetric stretching vibrations of CH_2_ (functional group of PVP), and the OH functional groups were established by the appearance of the band at 3360 cm^−1^. In Figure 1b–f, the result confirms the vibration of OH functional groups at 649 cm^−1^ and 3347 cm^−1^, respectively [34]. The PO_4_^3−^ vibration was confirmed by the prominent band at 573 cm^−1^, 842 cm^−1^ and 1083 cm^−1^, respectively; hence, the results confirmed the HAP presence in the fabricated nanofiber. The characteristic bands consequent to fabricated PVA and PVP (blended with HAP) are similar to the bands observed in Figure 1a, and in the polymeric network, the prominent bands for the PO_4_^3−^ and OH^−^ functional groups were found to be well defined.

### 3.2. XRD Analysis of HAP-PVA-PVP Nanofibers

**Figure 2:**
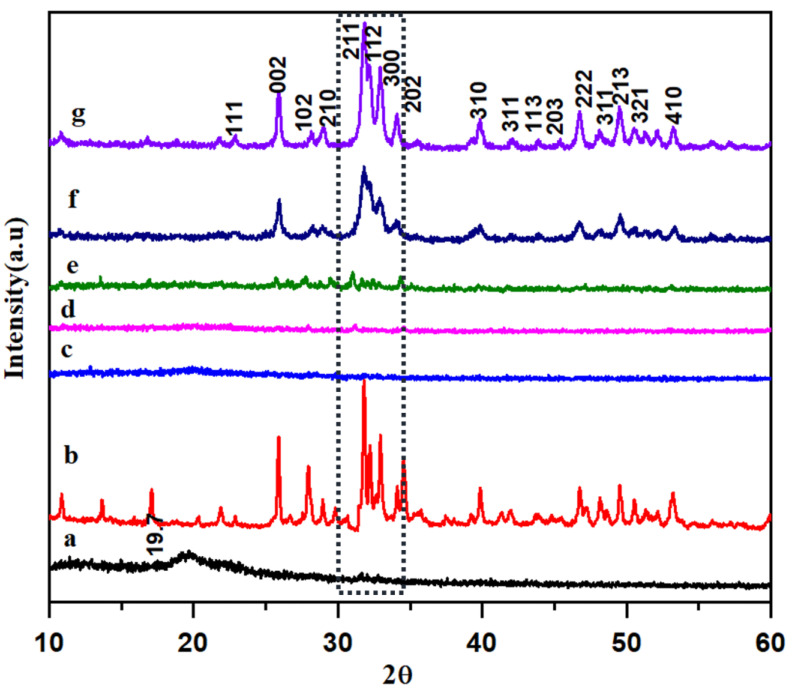
XRD analysis of (**a**) PVA-PVP; (**b**) pure HAP; (**c**) 5%; (**d**) 10%; (**e**) 15%; (**f**) 20%; and (**g**) 25% of HAP-PVA-PVP.

The XRD results of HAP-PVA-PVP composite nanofibers are displayed in Figure 2. The peaks at 2θ = 19.07 correspond to PVA- PVP. All the characteristic peaks of HAP at 2θ = 22.86 (111), 25.98 (002), 28.16 (102), 29.09 (210), 31.91 (211), 32.01 (112), 32.17 (300), 34.13 (202), 39.88 (310), 46.76 (222), 48.26 (312), 49.46 (213), 50.80 (321), and 51.25 (410) are well observed in the XRD spectrum [35]. The major triplet peaks at 2θ = 31.91 (211), 32.01 (112), and 32.17 (300) are found to be prominent with the increase in the concentration of HAP in the polymeric composite. With the addition of 5% HAP, the peak at 2θ = 19.07, which is related to the polymeric composite, was found to be decreased. The XRD spectrum with and without HAP seems to be a little amorphous because of the existence of the polymer in the scaffold. The intensity of the peaks was, thus, found to be increased by the incorporation of HAP in the PVA and PVP composite.

### 3.3. Thermal Stability of Composite Nanofibers

**Figure 3:**
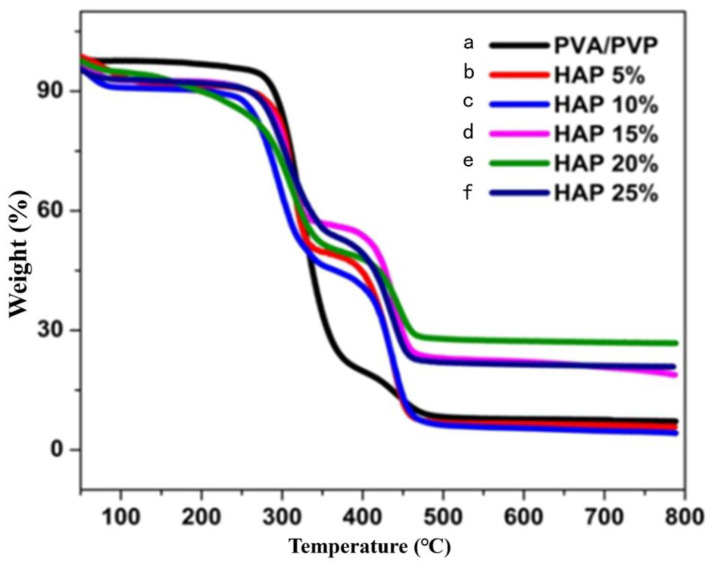
TGA analysis of (**a**) PVA and PVP; (**b**) 5%; (**c**) 10%; (**d**) 15%; (**e**) 20%; and (**f**) 25% of HAP-PVA-PVP.

**Figure 4:**
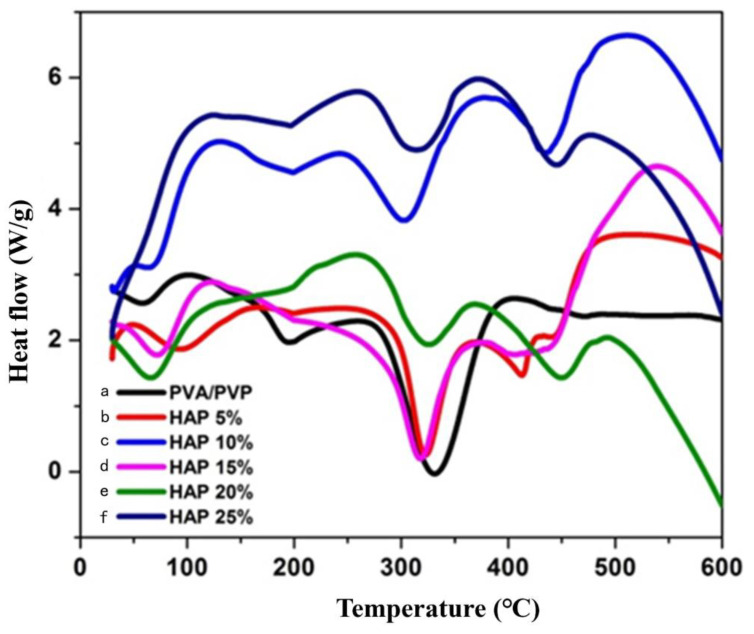
DSC analysis of (**a**) PVA and PVP; (**b**) 5%; (**c**) 10%; (**d**) 15%; (**e**) 20%; and (**f**) 25% of HAP-PVA-PVP.

The thermal stability of composite nanofibers with and without HAP was examined by thermal gravimetric analysis (Figure 3), and the results are shown in Table 2. The thermogram results reveal that the weight loss was observed in three stages, and the water loss attributed at 80–150 °C in the entire nanofibrous matrix was recognized as a first-stage loss. The weight loss of the second stage was established at around 340–360 °C because of the degradation of the polymeric side chain. The final stage of degradation was observed at 390–470 °C, which corresponds to the disintegration of the polymer backbone chain [36]. Thermal stability was due to the decrease in the decomposition rate of the nanofiber. Figure 4 and Table 3 display the melting temperature, glass transition temperature, and disintegration of the composite nanofibers. The melting temperature of the composite nanofibers in the absence of ceramics was observed at 194 °C with a glass transition temperature (TG) at 56.84 °C.

In addition, the melting temperature (TM) of composite nanofibers with 5–25% HAP was found to be decreased from 322 to 318 °C, and the glass transition temperature (TG) was found to be increased from 93.46 to 119.42 °C. Further, the decomposition peaks were found to be increased from 330 to 450 °C when increasing the HAP concentration, which proves the thermal stability of the nanofibers in the presence of HAP nanoparticles.

### 3.4. Morphology of Nanofibers Using SEM Analysis

**Figure 5:**
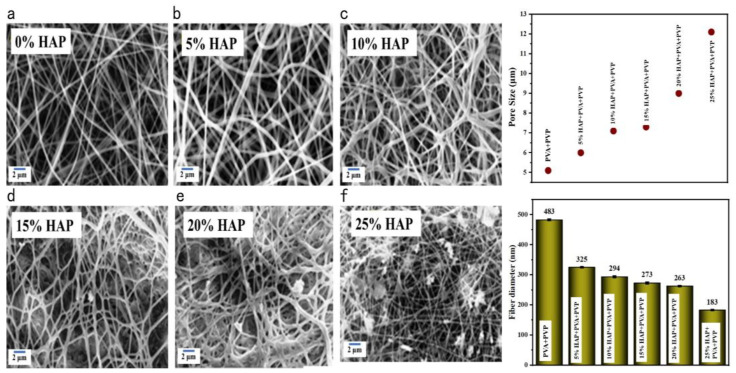
SEM analysis and pore distribution of (**a**) PVA-PVP; (**b**) 5%; (**c**) 10%; (**d**) 15%; (**e**) 20%; and (**f**) 25% of HAP-PVA-PVP.

Figure 5 shows the morphology and diameter of HAP-PVA-PVP composite nanofibers. The fiber diameter and pore size were calculated from the SEM images using Image J software (version no. 1.4.3.6.7) and are shown in Figure 5. With the increase in the concentration of HAP, the nanofiber diameter was found to be decreased along with the enhancement in pore size. The SEM image of nanofibers without HAP was found to be bead-free, with thick nanofiber. The morphology of nanofibers with 15 to 20% HAP and beaded and fiber size was reduced, and 25% HAP nanofibers were bead-free with thin fiber observed, owing to the incorporation of more HAP nanoparticles on the fibers.

From Figure 5 (pore diameter), it can be observed that the nanofiber without HAP has a diameter of 483 ± 52 nm, with a pore size range of 5.07 ± 0.61µm. Upon the addition of HAP into the nanofibers, the diameter of fiber was found to be influentially decreased from 325 nm to 183 nm. The pore size was found to be increased from 6 µm (5% HAP) to 12 μm (25% HAP), and this is advantageous in enriching the bioactivity of the fabricated nanofibrous scaffold and for more cell proliferation. The nanofibrous materials have several advantages due to their high surface area, high surface area-to-volume ratio, and high porosity, with a reduced fiber diameter, which make them promising materials for use as wound dressings and tissue-regenerative applications.

### 3.5. EDAX Analysis of HAP-PVA-PVP Nanofibers

**Figure 6:**
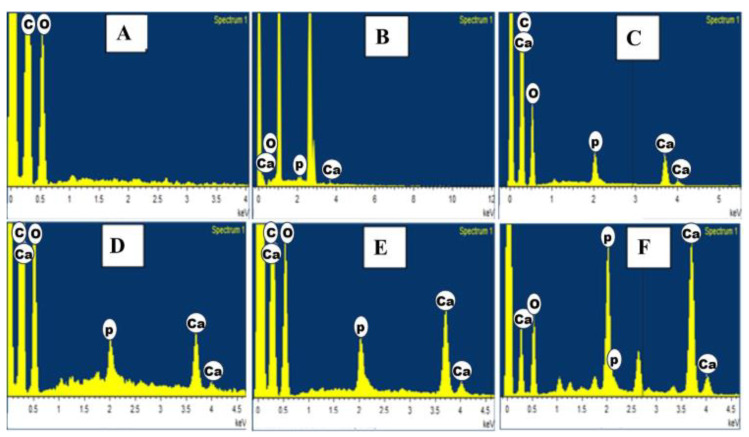
EDAX analysis of (**A**) PVA-PVP; (**B**) 5%; (**C**) 10%; (**D**) 15%; (**E**) 20%; and (**F**) 25% of HAP-PVA-PVP.

EDAX analysis is used to evaluate the atomic percentage of elements and also to determine the extent of mineral deposition attained in the fabricated nanofibers. The major component of cortical bone is hydroxyapatite, which contains bone minerals like calcium and phosphate [37]. Figure 6 reports the elemental composition of HAP from the EDAX spectra. It gives a Ca/P ratio of 1.64 for 5% of composite nanofibers, and the ratio was found to be increased when the HAP concentration increased up to 25% to a maximum Ca/P ratio of 1.67. The observed Ca/P ratio was found to be in close approximation to the stoichiometry of HAP [38,39]. Carbon and oxygen characteristic peaks are also observed in the EDAX analysis.

### 3.6. Morphology of Nanofibers Using TEM Analysis

**Figure 7:**
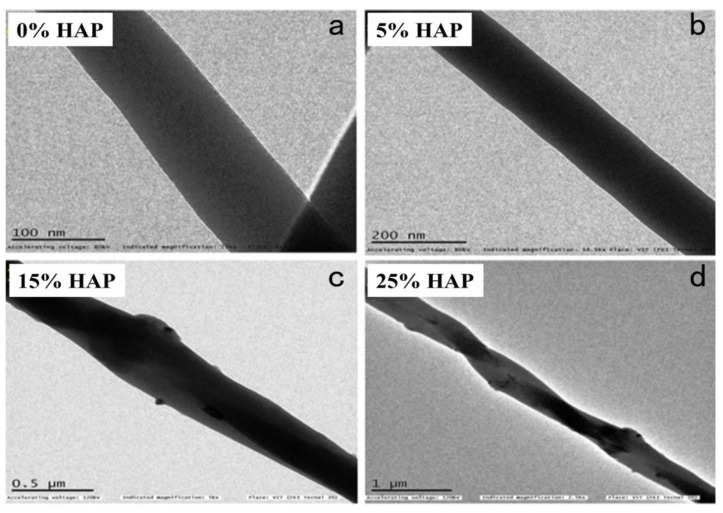
TEM analysis of (**a**) PVA and PVP (without HAP); (**b**) 5%; (**c**) 15%; and (**d**) 25% of HAP-PVA-PVP.

Figure 7 shows the morphology of nanofibers with various concentrations of HAP (0–25%) investigated via TEM analysis. The PVA-PVP composite nanofibers without HAP are smooth and bead-free, whereas the nanofiber consisting of 5% HAP shows the incorporation of HAP nanoparticles into the polymer matrix. The incorporation of HAP nanoparticles inside the polymer matrix was high when increasing the concentration of HAP, which is further evidenced by the decrease in the diameter of the nanofiber in accordance with SEM analysis. Further, an increase in the pore size and a corresponding decrease in the fiber diameter favor the cell growth for bone tissue engineering [40].

### 3.7. Bioactivity Analysis by SBF Immersion

**Figure 8:**
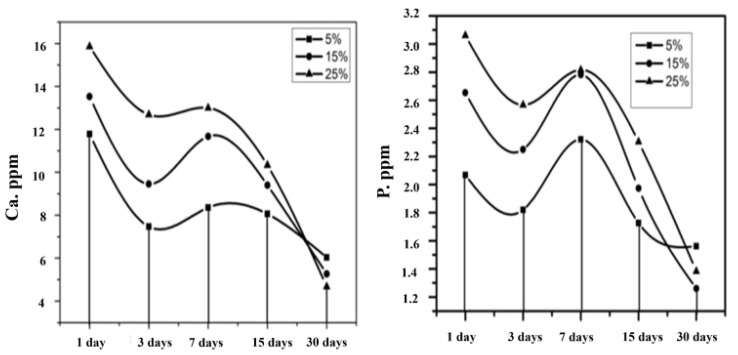
ICP-OES analysis of 5%, 15%, and 25% of HAP-PVA-PVP after SBF immersion at various intervals.

Figure 8 shows the result of the ICP-OES analysis with respect to the ionic concentration present in the SBF solution after the immersion of samples for various intervals. The composite nanofiber with 5%, 15%, and 25% of HAP was immersed in SBF solutions, and the solutions were subjected to ICP-OES analysis. The Ca and P ion interchange between the SBF and nanofibrous mat could be explained in three categories. The first category indicated that the Ca and P concentration decreased in SBF. The second category depicted a slight increase in the ionic concentrations or the termination of the reaction. The third category concluded with a continuous decrease in the Ca and P concentration [41]. The first phase leads to the Ca and P concentration decreasing for up to 3 days. This may be due to the Ca/P ion exchange from the SBF solution, forming a biolayer on the surface of the scaffold.

The second stage indicated a slight increase in the ionic concentration up to 5 to 7 days due to the establishment of equilibrium, with respect to ionic exchange between the nanofibrous matrix and SBF solution. Also, the SEM analysis revealed the formation of hydroxy-carbonate apatite (HCA) for 5% and 15% HAP-PVA-PVP, whereas 25% HAP-PVA-PVP showed a flower-like structure on the surface at 7 days of immersion.

The third category predicted a continuous decrease in the ionic concentration up to 15 to 30 days of immersion. This may be due to the precipitation of the calcium/phosphate layer, and this process is called the mineralization process between the SBF and scaffold. In this process, the ion enters into the pores and forms boundaries easily in the open pores of the reaction layer, and, hence, the degradation of the polymer is observed at the SBF interface [42].

**Figure 9:**
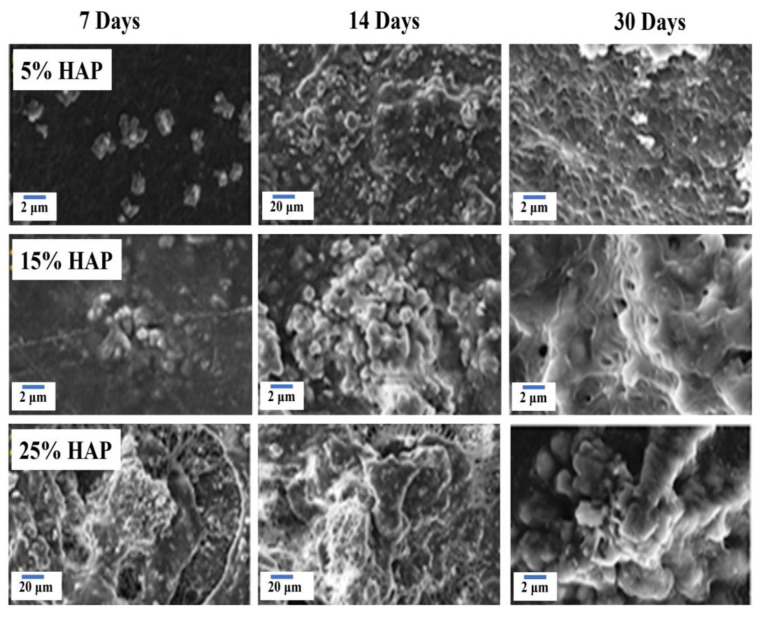
SEM analysis of SBF-immersed nanofiber for various intervals of 5%, 15%, and 25% of HAP-PVA-PVP.

In addition to this, the SEM (Figure 9) analysis proved the development of HCA over the specimen at 15–30 days of immersion. The surface was completely covered by the HCA layer, irrespective of the concentration of HAP in the polymer matrix. Further, from EDAX (Figure 9) results showed that the Ca/P ratio increases from 1.66 to 1.88 with various concentrations of HAP. Additionally, the above results were analyzed with XPS analysis to confirm the formation of an apatite layer over the specimen.

### 3.8. X-ray Photoelectron Spectroscopy (XPS)

**Figure 10:**
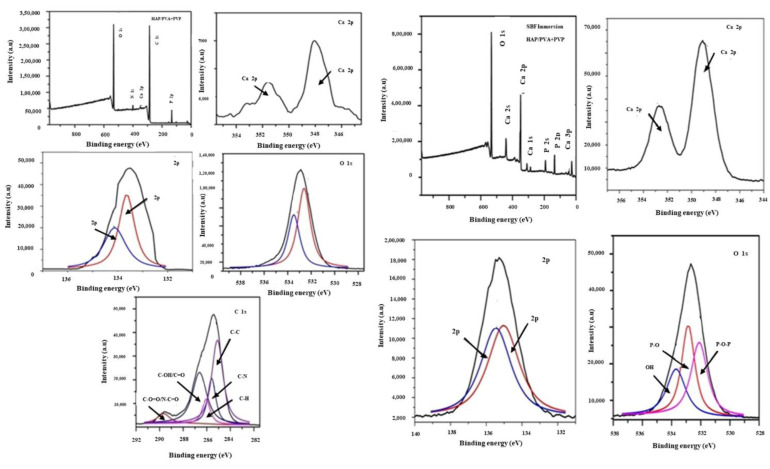
XPS full survey for before and after immersion of 25% HAP-PVA-PVP nanofiber and core level spectrum of Ca 2p, P 2p, O 1s, and C1s are mentioned.

Considering the outcomes observed in the above sections (FT-IR, XRD, SEM, and TEM analysis), the nanofiber matrix consisting of 25% of HAP was selected for SBF immersion and was further analyzed by XPS analysis to confirm the surface chemistry that occurred on the surface of the matrix. Figure 10 depicts the XPS analysis of the 25% HAP-PVA-PVP nanofiber composite, before and after SBF immersion. The full survey spectrum of the samples before and after SBF immersion had respective binding energy peaks of Ca 2p (348.1), P 2p (133), O 1s (532.9), C 1s (283.9), and N 1s (400.2), and Ca 2s (441.5), Ca 2p (349.1), Ca 3s (42.9), Ca 3p (26.1), P 2s (193.5), P 2p (135.2), and O 1s (532.9), respectively. Table 4 presents the binding energy and Ca/p ratio of the composite before and after immersion in SBF. In comparison with the SBF-immersed sample, the carbon and nitrogen peak in the polymer sample was found to be completely absent because of apatite formation on the nanofibrous surface.

The high-resolution XPS spectrum without SBF immersion had a core level of Ca (2P) in the 348.1 eV region and two deconvoluted peaks of Ca (2P1/2) and Ca (2P3/2) at 351.02 eV and 347.50 eV, respectively, for HAP. Another important core level of P (2P) was found to exist as two peaks, one at 134.14 eV, which corresponds to P (2P1/2), and the other at 133.66 eV, which corresponds to 2(2P3/2), respectively [43]. The O1s spectrum deconvoluted into two specific peaks at 532.56 and 531.27 eV, indicating the presence of “O” on the surface area as P-O and OH, respectively. As discussed earlier, the C1s region of PVA and PVP deconvoluted into five different binding energies at 289.67 eV, indicating the presence of O-C=O, C-C,-OH/C=O, C-N, C-H, elements with binding energies of 289.67 eV, 286.63 eV, 286.01 eV, 285.51 eV, and 284.75 eV, respectively.

It was found that the XPS spectrum of the SBF-immersed nanofiber matrix represents the well-developed binding energy of Ca/P and O with the absence of N and the reduction in C binding energy at 286.2 eV. The core level spectrum (Figure 10 after immersion) of Ca 3p, Ca 3s, P 2p, P 2s, Ca 2p, Ca 2s, O1s elements with corresponding binding energies of 26.133 eV, 42.93 eV, 135.2 eV, 193.5 eV, 349.1 eV, 441.50 eV, and 532.9 eV was found to be enhanced after SBF immersion [44]. Further, the core level O1s spectrum was found to be deconvoluted into three distinct peaks in the SBF-immersed nanofibrous matrix, owing to the existence of P-O-P, P-O, and OH binding energy at 529.7, 532.32, 533.27 eV, respectively. Hence, it was confirmed from the XPS survey that the polymer sample dissolved in SBF with the formation of an apatite layer by the increase in the Ca/P ratio from 1.66 to 1.71 (Table 4).

### 3.9. Hemolytic Assay

**Figure 11:**
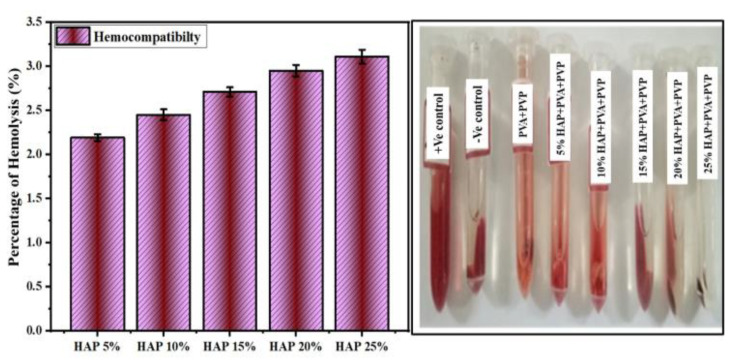
In vitro hemolytic assay for PVA and PVP and 5% to 25% of HAP-PVA-PVP nanofiber.

A hemocompatibility study was carried out for HAP-PVA-PVP with the HAP concentration ranging from 0 to 25% HAP in human blood to examine the compatibility of the samples with blood cells, and the results are shown in Figure 11. From Figure 11 and Table 5, it is observed that 5% of HAP-PVA-PVP is highly hemocompatible, and all the samples resulted in less than 5% hemolysis [45]. Both the polymer and HAP-PVA-PVP showed lower hemolytic values compared to a higher concentration of HAP in the PVA-PVP matrix,, which might be due to the leaching of Ca/P ions into the blood that can disrupt the membrane of RBC cells. However, the prepared nanofibers showed a lower percentage of hemolysis and, hence, the nanofibrous were extremely hemocompatible. Hence, the composite nanofibrous 25% HAP-PVA-PVP was selected for the in vitro biocompatibility evaluations.

### 3.10. Alkaline Phosphate Activity (ALP)

**Figure 12:**
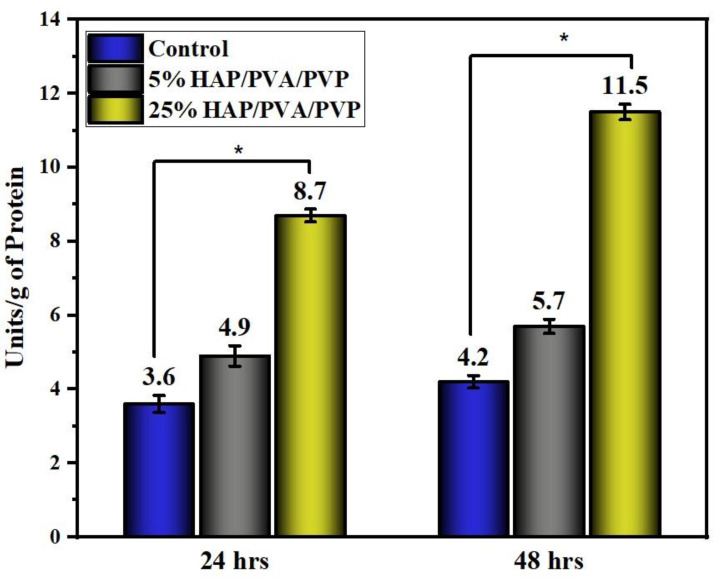
Alkaline phosphatase assay for 5% and 25% of HAP composite nanofibers. The (*) displays that the difference is statistically significant at *p* < 0.05 (data represent the mean ± standard deviation).

The in vitro biocompatibility assessment was carried out on 25% HAP-PVA-PVP due to its better hemolysis assay and better apatite formation on SBF immersion. Figure 12 shows the alkaline phosphatase assay for HAP-PVA-PVP with 5% and 25% HAP (for comparison, we incorporated 5% HAP-PVA-PVP). The composite nanofibers exhibited excellent osteoconductivity and biocompatibility, with zero toxicity due to the incorporation of HAP into the polymer matrix. The result shows a significant difference (i.e., overall *p*-value < 0.05) between the intervals (24 h and 48 h of incubation) and between the various concentrations of HAP (5% and 25%). Enhanced ALP activity was observed for 25% of HAP composite nanofibers, and the cells were able to promote an osteogenic function on the nanofiber mats compared to control and 5% of HAP-PVA-PVP nanofibers. These results proved that composite nanofibers stimulate osteoblast cell adhesion, proliferation, and differentiation,, which is further observed by confocal micrography (Figure 12).

### 3.11. Biocompatibility Assessment in Osteoblast Cell Lines

**Figure 13:**
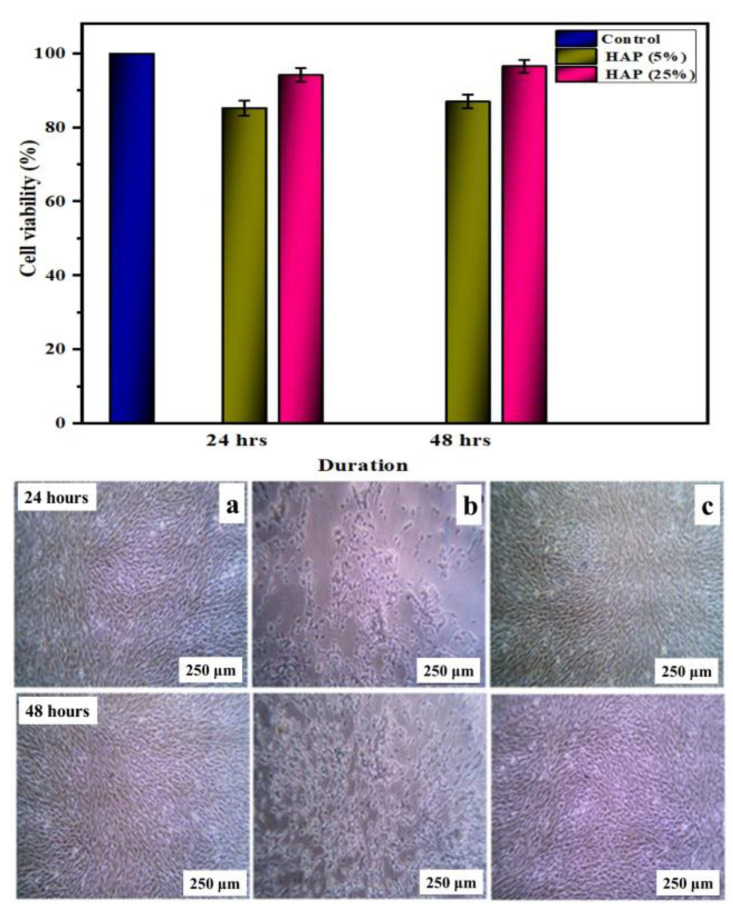
Biocompatibility results for (**a**) control (**b**), 5, and (**c**) 25% of HAP composite nanofibers.

Figure 13 reveals the MG-63 cell density response for HAP-PVA-PVP composite scaffolds, and the results are found to be excellent for both 24 h and 48 h of incubation. The microscopic images provided the adhered and elongated spindle-shaped morphology of the cells. The number of cells was found to be higher at 48 h due to cell proliferation caused by the presence of HAP in the matrix and the morphology provided by the polymeric scaffold [32]. In addition, the OD values significantly increased for the 25% HAP composite nanofiber compared to the 5% HAP concentration. Hence, this study confirmed that the fabricated composite consisting of 25% HAP-PVA-PVP has good cytocompatibility of MG-63 cells, and the images showed the presence of more active cells. No samples showed toxicity, but cell viability was observed for both the samples, and 25% HAP-PVA-PVP showed greater potential for cell attachment than other samples, as shown in Figure 13. Moreover, the composite scaffolds exhibited more than 90% of active cells with less toxicity, indicating the biocompatibility of the prepared matrix towards MG63 cells. Therefore, the scaffolds were proven to be non-toxic to human cells and have excellent cell viability performance, which can be considered for biomedical applications.

## 4. Conclusions

In this investigation, we successfully fabricated a nanofibrous matrix consisting of various concentrations of HAP using an electrospinning technique.

➢ The phase purity was confirmed by XRD diffraction, and a triplet peak corresponding to HAP was found to be well pronounced, with different concentrations of HAP. When the concentration of HAP increased, the fiber diameter was found to be decreasing, and the pore size of the nanofiber increased from 6 μm for the 5% HAP matrix to 12 μm for the 25% HAP matrix; these findings show an enrichment in the bioactivity of the fabricated nanofibrous scaffold when treated with SBF.
➢ By optimizing the parameters of the electrospinning of nanofibrous scaffolds, successful fabrication was obtained that exhibited a porous structure with the desired pore size and cumulative pore volume, which can help to increase the bioactivity of the fabricated matrix, and the results were further analyzed using SEM-EDAX and ICP-OES analysis.
➢ The XPS analysis confirmed the formation of apatite by the dissolution of the polymer during SBF immersion. The absence of “N” binding energy with the reduction in the “C” binding energy and the existence of P-O-P, P-O, and P-OH binding energy confirmed the formation of an apatite layer over the scaffold.
➢ In vitro hemocompatibility testing showed a lower percentage of hemolytic activity and, hence, proved that the matrices were more hemocompatible. In addition, both the alkaline phosphatase activity and MTT assay of the nanofibrous matrix on MG-63 cell lines exhibited good cytocompatibility, and from microscopic images, we observed that the cells adhered well, with an elongated spindle morphology. Hence, from the observed results, the fabricated multicomponent HAP-PVA-PVP nanofibrous scaffold could provide a potential matrix for bone-regenerative applications.

## Figures and Tables

**Table 1:** Composition ratios of PVA-PVP and HAP for electrospinning solution.

S.No	Concentration of PVA (%)	Concentration of PVP (%)	Concentration of HAP (wt%)
1	5	5	0
2	5	5	5 (0.5 g/10 mL)
3	5	5	10 (1.0 g/10 mL)
4	5	5	15 (1.5 g/10 mL)
5	5	5	20 (2.0 g/10 mL)
6	5	5	25 (2.5 g/10 mL)

**Table 2:** TGA analysis of (1) PVA and PVP; (2) 5%; (3) 10%; (4) 15%; (5) 20%; and (6) 25% of HAP-PVA-PVP.

S.No	Sample	Step 1 Weight Loss (°C)	Step 2 Weight Loss (°C)	Step 3 Weight Loss (°C)	% of Weight Loss
1	PVA-PVP	80	340	390	92.808
2	5% HAP-PVA-PVP	105	340	470	93.057
3	10% HAP-PVA-PVP	130	350	480	95.661
4	15% HAP-PVA-PVP	130	350	490	81.246
5	20% HAP-PVA-PVP	140	360	470	78.059
6	25% HAP-PVA-PVP	150	410	470	71.301

**Table 3:** DSC analysis of (1) PVA-PVP; (2) 5%; (3) 10%; (4) 15%; (5) 20%; and (6) 25% of HAP-PVA-PVP.

S.No	Sample	Tg	Tm	Decomposition Temperature
1	PVA-PVP	56.82	194	330
2	5% HAP-PVA-PVP	93.46	322	413
3	10% HAP-PVA-PVP	65.33	304	436
4	15% HAP-PVA-PVP	72.54	319	417
5	20% HAP-PVA-PVP	66.49	325	447
6	25% HAP-PVA-PVP	119.42	318	450

**Table 4:** XPS core level spectrum before and after immersion in SBF of 25% HAP-PVA-PVP nanofiber and Ca/ P ratio.

S.No	Sample	Ca 2p		P 2p		C1s	O1s	N
		2_P3/2_	2_P1/2_	2_P3/2_	2_P1/2_			
1	HAP-PVA-PVP (Before immersion)	347.50	351.02	133.66	134.14	(a) 284.75-(C-C) (b) 285.57-(C-N) (c) 286.63(C=O/COH) (d) 289.67(O-C=O)	532.32 533.27	400.02
2	HAP-PVA-PVP (After immersion)	349.10	352.63	134.94	135.65	286.2	532.56 533.27	Nil

**Table 5:** Hemo compatibility of the prepared electrospun nanofibers on human blood cells.

S.No	Sample	Hemolytic Ratio (% ±SD)
1	PVA-PVP	1.82 ± 0.045
2	5% HAP-PVA-PVP	2.19 ± 0.038
3	10% HAP-PVA-PVP	2.45 ± 0.062
4	15% HAP-PVA-PVP	2.71 ± 0.054
5	20% HAP-PVA-PVP	2.95 ± 0.065
6	25% HAP-PVA-PVP	3.11 ± 0.079

## Data Availability

Data will be available on request.
